# Influence of Gypsum Waste Utilization on Properties and Leachability of Fired Clay Brick

**DOI:** 10.3390/ma14112800

**Published:** 2021-05-24

**Authors:** Nur Jannah Abdul Hamid, Aeslina Abdul Kadir, Nurul Nabila Huda Hashar, Paweł Pietrusiewicz, Marcin Nabiałek, Izabela Wnuk, Marcek Gucwa, Paweł Palutkiewicz, Azini Amiza Hashim, Noor Amira Sarani, Amos Anak Nio, Norazian Mohamed Noor, Bartłomiej Jez

**Affiliations:** 1 Department of Water and Environmental Engineering, Faculty of Civil Engineering and Built Environment, Universiti Tun Hussein Onn Malaysia, Parit Raja 86400, Batu Pahat, Johor, Malaysia; jannahamid93@gmail.com (N.J.A.H.); nurulnabilahudahashar@gmail.com (N.N.H.H.); aziniamizaa@gmail.com (A.A.H.); nramira1987@gmail.com (N.A.S.); amosanaknio@gmail.com (A.A.N.); 2 Micropollutant Research Centre (MPRC), Universiti Tun Hussein Onn Malaysia, Parit Raja 86400, Batu Pahat, Johor, Malaysia; 3 Center of Excellence Geopolymer and Green Technology (CEGeoGTech), University Malaysia Perlis, Arau 02600, Perlis, Malaysia; norazian@unimap.edu.my; 4 Department of Physics, Częstochowa University of Technology, 42-201 Częstochowa, Poland; pawel.pietrusiewicz@pcz.pl (P.P.); nmarcell@wp.pl (M.N.); izabela.wnuk@pcz.pl (I.W.); bartek199.91@o2.pl (B.J.); 5 Department of Technology and Automation, Faculty of Mechanical Engineering and Computer Science, Częstochowa University of Technology, 42-201 Częstochowa, Poland; mgucwa@spaw.pcz.pl (M.G.); palutkiewicz@ipp.pcz.pl (P.P.)

**Keywords:** fired clay brick, gypsum waste, properties, leaching test, synthetic precipitation leaching procedure

## Abstract

Wastewater treatment activities in the chemical industry have generated abundant gypsum waste, classified as scheduled waste (SW205) under the Environmental Quality Regulations 2005. The waste needs to be disposed into a secure landfill due to the high heavy metals content which is becoming a threat to the environment. Hence, an alternative disposal method was evaluated by recycling the waste into fired clay brick. The brick samples were incorporated with different percentages of gypsum waste (0% as control, 10, 20, 30, 40 and 50%) and were fired at 1050 °C using 1 °C per minute heating rate. Shrinkage, dry density, initial rate of suction (IRS) and compressive strength tests were conducted to determine the physical and mechanical properties of the brick, while the synthetic precipitation leaching procedure (SPLP) was performed to scrutinize the leachability of heavy metals from the crushed brick samples. The results showed that the properties would decrease through the incorporation of gypsum waste and indicated the best result at 10% of waste utilization with 47.5% of shrinkage, 1.37% of dry density, 22.87% of IRS and 28.3% of compressive strength. In addition, the leachability test highlighted that the concentrations of Fe and Al was significantly reduced up to 100% from 4884 to 3.13 ppm (Fe) and from 16,134 to 0.81 ppm (Al), respectively. The heavy metals content in the bricks were oxidized during the firing process, which signified the successful remediation of heavy metals in the samples. Based on the permissible incorporation of gypsum waste into fired clay brick, this study promised a more green disposing method for gypsum waste, and insight as a potential towards achieving a sustainable end product.

## 1. Introduction

Waste is defined as the substance prescribed to be the scheduled waste or any other matter in the form of a solid, semi-solid, liquid, vapor, or gas [1]. Additionally, it could be emitted, discharged or deposited onto the environment in such a volume, composition or manner to cause pollution. In Malaysia, 1.6 million metric tons of industrial sludge is produced annually, while 0.81 million metric tons are disposed at sanitary landfills [1]. Scheduled wastes are the categories of waste listed under the First Schedule of Environmental Quality Regulations 2005 [2]. The categories of the scheduled waste are classified as environmental hazardous waste due to the toxic and high-risk nature of a particular type of waste. Gypsum waste that arose from the chemical industry was classified as the waste containing principally inorganic constituents, which may comprise metals and organic materials.

Gypsum waste becomes a very serious environmental issue as the common disposal methods applied involve landfilling and burning in the incinerator. This situation affects people’s health and sanitary issues. Gypsum waste was banned from being disposed in a normal landfill as it was mixed with other biodegradable waste which led to hydrogen sulfide gas emission through microbial action [3]. In addition, hydrogen sulfide is toxic, colorless and flammable and has distinct foul odor of rotten eggs that could cause breathing difficulties, discoloration of the skin and eye irritation [4]. Nevertheless, the current limited landfill has urged the search for an alternative disposal method since the existing landfills can no longer accommodate the disposal of gypsum waste.

Recently, various measures have been carried out to create more environmentally friendly and economic products with similar original resilience [5,6]. The products will be embedded with residual waste from industry or residential waste. Therefore, the incorporation of gypsum waste into fired clay brick is an attempt to save the space for landfill, the cost of disposal management as well the environment [7]. Simultaneously, the rapid growth in the construction sector has led to the demand for building materials such as fired clay bricks due to its properties. Hence, this study was conducted to investigate the properties and leaching behavior of fired clay bricks incorporated into gypsum waste. Besides, this study also focused on both properties and leachability where most previous studies only discussed the properties of a particular product.

## 2. Materials and Methods

### 2.1. Raw Materials and Their Preparation

**Figure 1:**
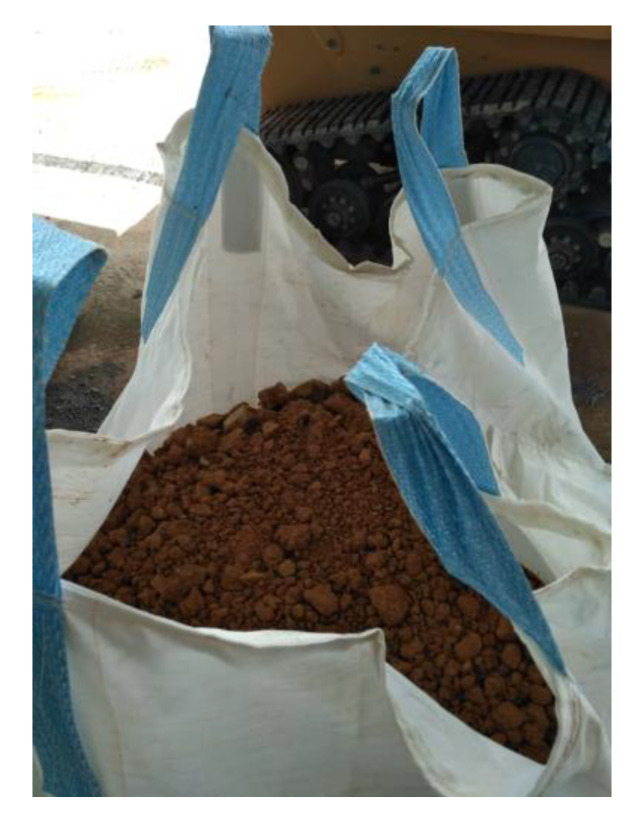
Gypsum waste from chemical industry.

In this study, gypsum waste was obtained from a chemical industry located in Johor Bahru, Johor, Malaysia. The gypsum waste was in sludge form, as shown in Figure 1, while the clay soil was collected from a quarry in Batu Pahat, Johor. Both of the raw materials were dried in the oven (Memmert, +300 °C, Schwabach, Germany) at 105 °C for 24 h to remove the water content before being ground in order to obtain the uniform size of the particles to yield a homogeneous mixture of brick. The gypsum waste and clay soil were kept in a closed container to avoid any contaminants getting into them before being used.

### 2.2. Chemical and Geotechnical Properties

The chemical characterization of the raw materials was determined by using X-ray fluorescence (XRF, Philips, PW1840, Malvern, UK). Geotechnical properties of the raw materials used were determined by conducting the Atterberg Limit test, specific gravity test and standard proctor test. The Atterberg Limit and specific gravity were investigated in accordance the BS-1377-2 [8] and the standard proctor test was based on BS-1377-4 [9]. The optimum moisture content for control brick and clay-gypsum waste bricks was determined to produce a good quality of brick.

### 2.3. Methods Used for Brick Samples Preparation

There were two types of bricks manufactured which consist of control bricks and clay-gypsum waste bricks. The control brick contained 100% clay soil without gypsum waste incorporation, while the clay-gypsum waste brick was designed with different gypsum waste content (10, 20, 30, 40 and 50%), as shown in Table 1. The processes to manufacture the brick samples was started by mixing the clay soil and gypsum waste with a predetermined amount of water using a mechanical mixer with a 10 L capacity (Hobart Mechanical Mixer, South Ridge Ave., Troy, OH, USA). After the mixture was homogeneously prepared, the samples were put into a mold with the dimension of 215 mm × 102.5 mm × 65 mm and were compacted at the pressure of 2000 psi (Eco Global Technology, Brick machine, Johor Bahru, Malaysia). Then, the samples were dried at room temperature for 24 h with another 24 h in the oven at the temperature of 105 °C [10]. The drying process was carried out subsequently to prevent disintegration due to the rapid loss of moisture content within the brick samples.

**Figure 2:**
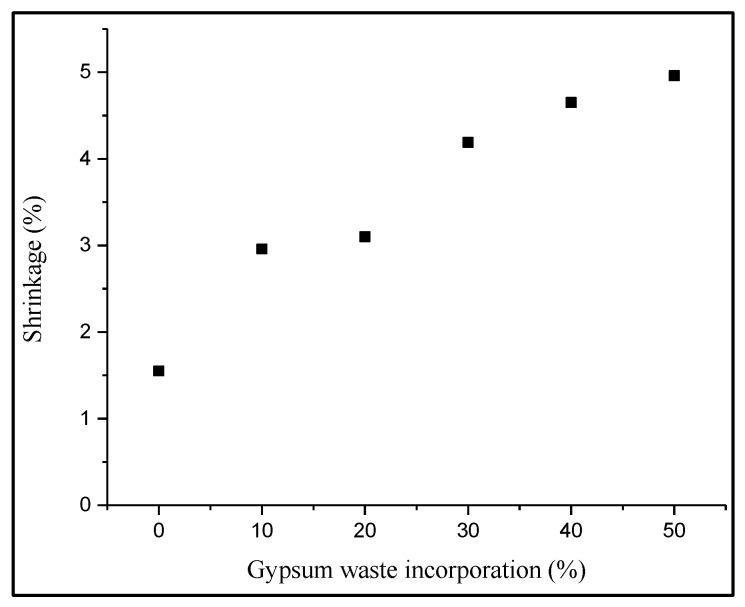
Effect of gypsum waste utilization on the shrinkage of fired clay brick.

Next, as shown in Figure 2, by using the heating rate of 1 °C/min, the samples were gradually fired in the furnace up to a firing temperature of 1050 °C for two hours [11]. The selected firing temperature and heating rate were closely related to the current practice in the brick industry. Besides that, the exemplary firing temperature reported in a few studies indicated that 1050 °C could achieve the vitrification phase at an optimal level which contributed to the better particle bonding, leading to the high strength of brick samples produced [12,13,14]. Thereafter, all brick samples were tested for physical and mechanical properties including shrinkage, dry density, initial rate of suction (IRS) [15] and compressive strength according to the British Standard (BS 3921: 1985) [16].

The leaching behavior of the brick samples was tested according to the synthetic precipitation leaching procedure (SPLP) to determine the concentration of heavy metals as, described in Method 1312 [17]. The leaching test was conducted to not exceed the regulatory threshold limit set by the United State Environment Protection Agency. The SPLP was designed to stimulate the condition of brick samples when being exposed to acid rain.

The samples were prepared in a screw-capped polyethylene bottles which were filled with crushed samples and leaching fluid at the ratio of 1:20. The particle size was required to pass through 9.5 mm standard sieve and the extraction fluid employed was a pH 4.2 solution consisted of sulfuric acid/nitric acid (H_2_SO_4_/HNO_3_), which were mixed carefully. The bottles were rotated by using a rotary agitation holder for about 18 h at 30 rpm. Then, the mix underwent the infiltration process using 0.7 µm glass fiber filters (Whatman, Maidstone, Kent, UK) to filter rough residues before being analyzed with inductive coupled plasma mass spectrometry (ICP-MS, PerkinElmer Elan9000, Shelton, CT, USA). The results obtained were compared to the United States Environmental Protection Agency (USEPA) standard [17].

## 3. Results and Discussion

### 3.1. Properties of Raw Materials

X-ray fluorescence (XRF) was used to determine the main composition of the raw materials used, which were clay and gypsum waste, as shown in Table 2. The highest element contents in the clay soil were SiO_2_ and Al_2_O_3_ with 40.8% and 38.70%, respectively, while CaO had the lowest concentration, which was only 0.10%. The results were consistent with previous studies that showed the high composition of silica and alumina in raw materials can enhance the refractoriness of the bricks, thus, improved the mechanical properties as well [18,19]. Meanwhile, for gypsum waste, the highest concentration were CaO and SO_3_ at 29.57% and 29.56%, respectively. Concurrently, the valuable components of calcium sulfate in gypsum had the potential to be used as the binder for waste sludge treatment. The presence of CaO, MgO and Na_2_O in gypsum waste were higher than in the clay soil were reported as fluxing agents that can decrease the firing temperature, hence, lowering the energy consumption used during firing stage [20,21]. Moreover, the amount of Fe_2_O_3_ in both of the raw materials were quite similar, where it resembled the reddish color of bricks after the firing process.

The geotechnical properties of the clay soil were tabulated as in Table 3. The liquid limit and plasticity for the clay soil was 29.3% and 13.1%, respectively. In order to produce a good quality and fair properties of bricks, the limit value for the plastic limit value was required to be in the range of 12 to 22%, while for the plasticity index, the range was 7 to 18%, as recommended in the associated literature on small-scale brickmaking [22]. Clay soil, that has a well-known flexibility to bind with another material, could be a great potential in producing clay-gypsum waste brick with a medium degree of plasticity and being classified as silt clay in this study [23].

### 3.2. Shrinkage of Manufactured Bricks

Shrinkage occurs when the loss of capillary water during the drying and firing processes causes contraction in the hardened mixture. The factors that influenced the amount of shrinkage were basically the characteristics of the materials mixed, the proportion of the mixture, manufacturing processes, moisture content and dry condition applied [24]. The shrinkage of manufactured fired clay brick samples is presented in Figure 2. The results showed that the samples with 50% gypsum waste incorporation had the highest value of shrinkage with 4.96%, followed by samples with 40% gypsum with the value of 4.65%. This showed that by increasing the percentage of gypsum waste incorporation, the shrinkage of the fired clay brick would increase. The 10% gypsum waste utilization demonstrated 47.5% shrinkage of the control brick value. The similar trend of the results was also recorded in a previous study which incorporated waste sludge into clay bricks. The study reported that the least amount of shrinkage must be below 8% to be regarded as a good quality of bricks [25]. Moreover, the shrinkage value of gypsum brick was higher due to water demand during the mixing process, which was more compared to the control brick. Although the shrinkage value increased, the gypsum brick still complied with the preferable shrinkage properties up to 20% with the value of 3.1% [26]. Additionally, the drying and firing stage had caused the loss of water particles in the bricks and affected the shrinkage behaviors relatively [27].

### 3.3. Dry Density of Manufactured Bricks

**Figure 3:**
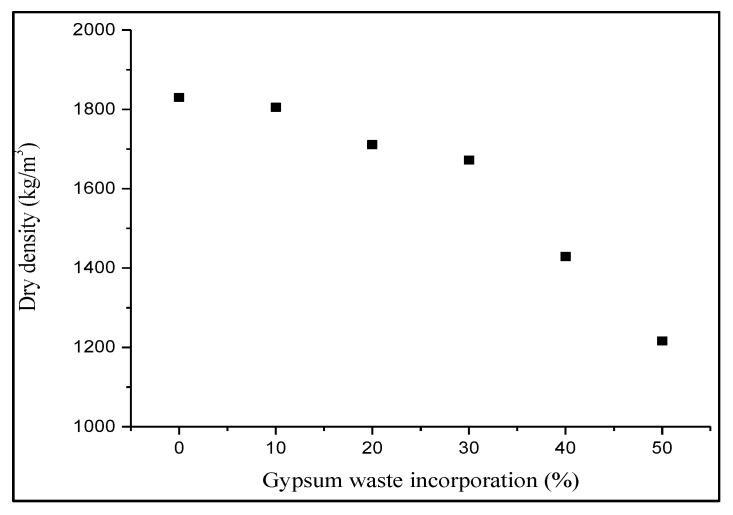
Effect of gypsum waste utilization on the dry density of fired clay brick.

Based on Figure 3, the dry density of the fired clay brick manufactured varies from 1830 to 1216 kg/m^3^ in different percentages. The 10, 20, 30 and 40% gypsum brick recorded 1805, 1711, 1672 and 1429 kg/m^3^ of dry density values, respectively, which decreased gradually with the increase in gypsum waste incorporation. The same results were reported where the weight of the bricks was reduced when the waste content was increased [28,29]. Meanwhile, the control brick had the highest dry density with 1830 kg/m^3^ whilst the lowest density was 50% of gypsum brick with the value of 1216 kg/m^3^. The particle density of the bricks decreased when the sludge waste was added proportionally, which is supported by [28,29]. This trend related to the specific gravity (SG) value of the clay soil, which was higher than gypsum waste and had affected the density of the bricks since more water was absorbed into the larger pores within the brick bodies. Therefore, a lightweight brick was produced with the increasing gypsum waste content. Moreover, the gypsum waste tended to get burnt away during the firing process due to high temperature exposure, consequently creating pores [30]. Although gypsum bricks had a lower dry density than the control brick, the density values still indicated that all the samples complied with the standard density requirement. According to [30], gypsum has the potential to bind soil particles and could enhance the strength of soil mixtures, especially in a dry environment. Despite the nature of gypsum which was soluble in water, the firing process helped in removing three-quarters of the water molecules by evaporation and the formation of stable elements encapsulated in the bricks.

### 3.4. Initial Rate of Suction (IRS) of Manufactured Bricks

**Figure 4:**
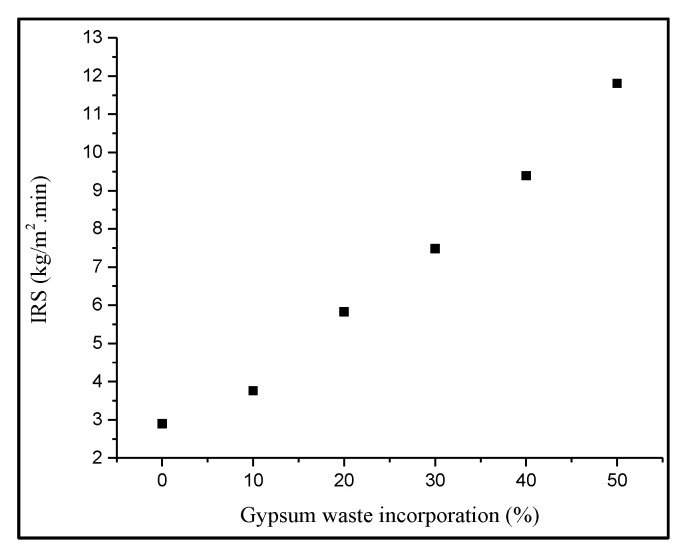
Effect of gypsum waste utilization on the initial rate of suction of fired clay brick.

The initial rate of suction (IRS) value represents the ability and the potential performance of the brick in laying and durability. Based on Figure 4, the control brick showed the lowest value of IRS compared to the gypsum brick with the value of 2.9 kg/m^2^.min. The highest IRS value was from the 50% gypsum brick with 11.81 kg/m^2^.min and followed by the 40, 30, 20 and 10% gypsum brick with value of IRS at 9.39, 7.48, 5.83 and 3.76 kg/m^2^.min, respectively. The results indicated that the IRS values increased when the percentages of gypsum waste increased due to the interconnected pores, voids and capillaries that appeared in the bricks. The results recorded a similar trend in the previous study which supported the suction rate was increased as the waste content increased [31]. According to BS EN 771-1 [32]. the value of IRS should be lower than 2 kg/m^2^.min, but unfortunately, all the bricks showed higher values which were more than the acceptable limit. Gypsum bricks showed the highest value of IRS as gypsum waste was prone to be burnt away during the firing process due to the high temperature, hence resulting in pore development in the brick. Bricks with high porosity tended to absorb more water compared to the brick with low porosity and could lead to volume changes and cause cracking to the bodies of the bricks [33]. Since there is limited specific guidance for IRS requirements, the gypsum brick could still be useful in construction works but must be wetted earlier from three hours to 24 h before being laid [34].

### 3.5. Compressive Strength of Manufactured Bricks

**Figure 5:**
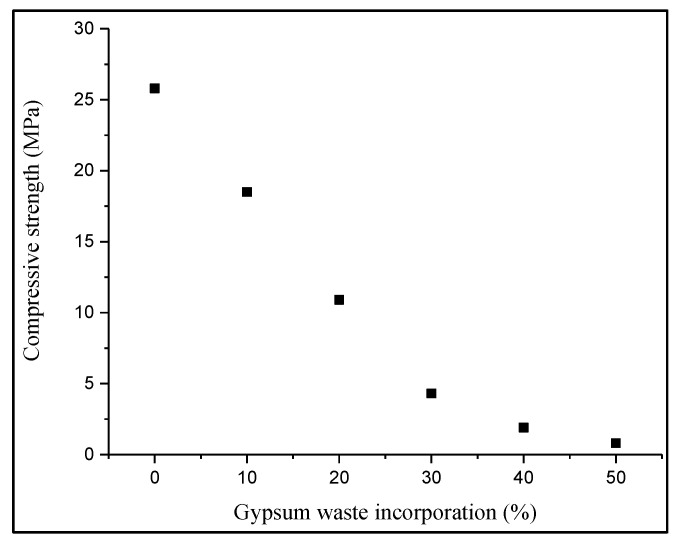
Effect of gypsum waste utilization on the compressive strength of fired clay brick.

According to Figure 5, the results showed that the highest compressive strength was achieved by the control brick with 25.8 MPa, followed by the 10 and 20% gypsum brick at 18.5 and 10.9 MPa, respectively. In addition, the 30, 40 and 50% gypsum bricks with the values of 4.3, 1.9 and 0.8 MPa, respectively, did not comply with the standard of BS 3921: 1985 where the minimum requirement of compressive strength of the brick should not be less than 7.0 MPa. The compressive strength of the gypsum brick was lower due to the high porosity and high IRS value compared to control bricks [35]. Based on the results, the increasing amount of gypsum waste incorporation into fired clay brick lowered the density of the gypsum bricks, consequently decreasing the compressive strength. Additionally, a previous study investigated showed that a weak inter-particulate within the manufactured brick occurred when the clay soil burnt during the firing process [36]. Besides that, the previous study claimed the same trend that the disintegration of the particles in the bricks during the firing stage had weakened the clay-waste composite caused by the presence of pores [37]. Apart from that, for gypsum brick with 10 and 20% gypsum waste incorporation fulfilled the requirement for the non-load bearing applications, which should not be less than 5 N/mm^2^ according to BS 3921:1985.

### 3.6. Leachability of Fired Clay Brick

**Figure 6:**
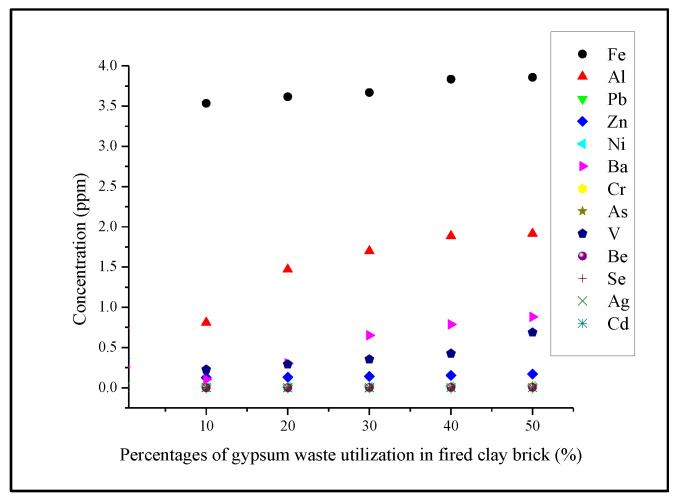
Effect of gypsum waste utilization on the leachate concentration of heavy metals in fired clay brick.

The leachability of heavy metals in the samples was carried out on crushed samples for short-term testing and the results are presented in Table 4. The highest concentration of heavy metal in gypsum brick was iron (Fe) with 3.13, 3.61, 3.66, 3.83 and 3.85 ppm for 10, 20, 30, 40 and 50%, respectively. The Fe concentration in the gypsum brick also showed a huge difference compared to the control brick, which was 0.033 ppm, which represented the permeability and durability of bricks [38]. Meanwhile, the highest concentration of aluminum (Al) was showed in the gypsum brick at 50% with 1.91 ppm followed by the gypsum brick at 40% with 1.88 ppm. The results were consistent with the studies conducted previously that stated during the firing process, the heavy metals were oxidized, which reduced the level of the toxicity in the bricks [38]. In addition, the immobilization of heavy metals occurred at a high firing temperature [39,40]. However, all the heavy metals were in reliance and the gypsum bricks were complied with the standard concentration limits. From the SPLP results, the pH of leachate mixture was considered as one of the factors that contributed to the lower heavy metals amount in the leachate, as shown in Figure 6. The values were below the USEPA standard; thus, the incorporation of gypsum waste into fired clay brick could be an alternative to reutilize the gypsum waste in fired clay brick. Furthermore, the utilization of gypsum as a building material is gaining more attention as it is one of the most environmentally friendly binders [41].

## 4. Conclusions

From the study, the properties and leachability of fired clay bricks incorporated with gypsum waste were determined. Based on the results, the dry density and compressive strength of gypsum brick compatibly decreased when the amount of gypsum waste incorporation increased compared to the control brick. This happened as the utilization of gypsum waste in fired clay brick increased the quantity of pores and lowered the density of brick, which caused the fired clay brick to easily break. However, the gypsum brick with up to 20% still fulfilled the minimum requirement of the standard, but not for the 30, 40 and 50% gypsum bricks. Meanwhile, the values of shrinkage and IRS were increased by increasing the amount of gypsum waste into the fired clay brick. Apparently, only up to 20% of the gypsum waste incorporation produced fired clay brick with fair properties as stated in the standard. Nevertheless, the best result was demonstrated by 10% of waste utilization with 47.5% of shrinkage, 1.37% of dry density, 22.87% of IRS and 28.3% of compressive strength shown by the adequate properties possessed.

The leachability test highlighted that the concentrations of the heavy metals were significantly reduced after the firing process. The findings from SPLP also showed that the concentrations of the heavy metals in all crushed samples manufactured did not exceed the regulatory limit specified by USEPA. This indicated that incorporating gypsum waste into fired clay brick could produce a new alternative disposal method for gypsum waste generated, which was highly recommended to be used as the building material. In a nutshell, this effort is an effective solution in enhancing the lifespan of the landfill and possibly reducing the pollution of the environment. Further investigation on the emissions released during the firing process of manufacturing the gypsum bricks shall be conducted in the subsequent study.

## Figures and Tables

**Table 1:** Design of mixtures for manufactured fired clay brick.

Sample	Gypsum Waste (%)	Clay Soil (%)	Gypsum Waste (kg)	Clay Soil (kg)	Total Mass (kg)	Water (mL)
Control brick	0	100	0.00	2.80	2.80	475
Gypsum brick	10	90	0.20	2.60	2.80	515
	20	80	0.47	2.33	2.80	550
	30	70	0.81	1.99	2.80	585
	40	60	1.25	1.55	2.80	650
	50	50	1.88	0.92	2.80	695

**Table 2:** XRF of clay soil and gypsum waste.

No	Parameter	Concentration (wt%)	
		Clay Soil	Gypsum Waste
1	CaO	0.10	29.57
2	SO_3_	0.27	29.56
3	Al_2_O_3_	38.70	16.13
4	SiO_2_	40.8	8.86
5	MgO	0.36	7.82
6	Fe_2_O_3_	5.41	4.88
7	Na_2_O	0.47	2.39
8	P_2_O_5_	-	0.39
9	MnO	-	0.15
10	K_2_O	0.22	0.11
11	Cl	-	418.00 mg/kg
12	TiO_2_	1.05	344.00 mg/kg
13	ZnO	-	111.00 mg/kg
14	Cr_2_O_3_	-	56.00 mg/kg

**Table 3:** Geotechnical properties.

Properties	Clay Soil
Specific gravity	2.6 (clay soil), 2.5 (gypsum waste)
Liquid limit (%)	29.3
Plastic limit (%)	16.2
Plasticity index (%)	13.1
Degree of plasticity	Medium Plastic
Type of soil	Silty clay or clayey silt

**Table 4:** Heavy metals in fired clay brick samples by using SPLP.

Heavy Metals	Concentration (ppm)						USEPA
	Control Brick	Gypsum Brick					
		10%	20%	30%	40%	50%	
Cu	0.0137	0.0011	0.0029	0.0123	0.0168	0.0173	100
Fe	4.3290	3.1350	3.6169	3.6689	3.8348	3.8585	NA
Al	0.7670	0.8110	1.4706	1.6990	1.8863	1.9160	NA
Pb	0.0271	0.0019	0.0025	0.0032	0.0041	0.0056	5
Zn	0.3670	0.1270	0.1309	0.1415	0.1540	0.1705	500
Ni	0.0024	0.0089	0.0092	0.0103	0.0166	0.0189	1.34
Ba	0.2540	0.1040	0.3030	0.6520	0.7880	0.8810	100
Cr	0.0113	0.0042	0.0058	0.0072	0.0114	0.0160	5
As	0.0027	0.0065	0.0087	0.0095	0.0108	0.0174	5
V	0.2060	0.2280	0.2930	0.3550	0.4260	0.6880	NA
Be	0.0005	0.0002	0.0003	0.0034	0.0043	0.0056	NA
Se	0.0027	0.0027	0.0035	0.0049	0.0057	0.0084	NA
Ag	0.0027	0.0028	0.0022	0.0022	0.0028	0.0025	NA
Cd	0.0011	0.0003	0.0002	0.0003	0.0007	0.0005	1

NA stand for Not Applicable.

## Data Availability

The data presented in this study are available on request from the corresponding author.
